# Sildenafil Can Affect Innate and Adaptive Immune System in Both Experimental Animals and Patients

**DOI:** 10.1155/2017/4541958

**Published:** 2017-02-20

**Authors:** Monika Kniotek, Agnieszka Boguska

**Affiliations:** Department of Clinical Immunology, Institute of Transplantology, Medical University of Warsaw, Nowogrodzka 59, 02-006 Warsaw, Poland

## Abstract

Sildenafil, a type 5 phosphodiesterase inhibitor (PDE5-I), is primarily used for treating erectile dysfunction. Sildenafil inhibits the degradation of cyclic guanosine monophosphate (cGMP) by competing with cGMP for binding site of PDE5. cGMP is a secondary messenger activating protein kinases and a common regulator of ion channel conductance, glycogenolysis, and cellular apoptosis. PDE5 inhibitors (PDE-Is) found application in cardiology, nephrology, urology, dermatology, oncology, and gynecology. Positive result of sildenafil treatment is closely connected with its immunomodulatory effects. Sildenafil influences angiogenesis, platelet activation, proliferation of regulatory T cells, and production of proinflammatory cytokines and autoantibodies. Sildenafil action in humans and animals appears to be different. Surprisingly, it also acts differently in males and females organisms. Although the immunomodulatory effects of PDE5 inhibitors appear to be promising, none of them reached the point of being tested in clinical trials. Data on the influence of selective PDE5-Is on the human immune system are limited. The main objective of this review is to discuss the immunomodulatory effects of sildenafil in both patients and experimental animals. This is the first review of the current state of knowledge about the effects of sildenafil on the immune system.

## 1. Introduction

Phosphodiesterases (PDEs) are critical components in the cyclic adenosine monophosphate/protein kinase A (cAMP-PKA) and the cyclic guanosine monophosphate/protein kinase G (cGMP-PKG) signaling pathways. Phosphodiesterases decompose the cyclic nucleotides, cAMP and cGMP, to inactivate 5′-AMP and 5′-GMP, respectively. PDE activity is tightly coupled with the activity of adenyl cyclases (AC) and guanyl (GC) which synthesize these cyclic nucleotides: cAMP and cGMP. The cyclic nucleotide-dependent protein kinases (PKA and PKG) control the functional cellular responses such as intracellular calcium level, inflammation, cell proliferation, and transcription. Therefore, cAMP and cGMP are considered as potential new therapeutic targets [1, 2]. The inhibition of action of PDE results in increased level of cyclic nucleotides.

Several PDEs have been identified and characterized based on their molecular sequence, kinetics, regulation, and pharmacological characteristics. These enzymes were divided into 11 families (PDE1–PDE11) [3, 4]. PDEs 1, 2, 3, 10, and 11 have affinity to cAMP and cGMP and PDEs 4, 7, and 8 hydrolyze cAMP only, while PDEs 5, 6, and 9 exhibit specificity for cGMP. Increased activity of PDEs has been implicated in a number of clinical conditions including erectile dysfunction (PDE5), cardiovascular diseases (PDEs 3, 4, and 5), pulmonary inflammatory conditions (PDE4), autoimmune diseases (PDEs 3 4, 5, and 7), and cognition and memory disorders (PDEs 1, 2, 3, 4, 7, 9, and 10 A) [5–7].

### 1.1. Phosphodiesterases Structure

All PDEs are composed of 3 main domains: the catalytic core (highly conserved), the regulatory N-terminus, and the carboxyl C-terminus. N-terminal domains are responsible for regulation and subcellular localization of PDEs. They contain domains involved in ligand binding, PDE oligomerization, kinase recognition, and phosphorylation. The N-terminal domains are flanked by the catalytic core. The catalytic c-domain of PDEs possesses an active pocket composed of 3 helical subdomains: the N-terminal cyclic-fold region, the linker region, and the C-terminal helical bundle. A deep hydrophobic pocket is formed at the interface of the three subdomains. The hydrophobic pocket is composed of four subsites: a metal binding site (M), a core pocket (Q pocket), hydrophobic pocket (H pocket), and a lid-region (L region). At the bottom site is a metal binding domain which probably contains zinc or magnesium ions. Stabilization of the enzyme structure and activation of hydroxide are the presumable roles of metal ions [7, 8]. Substrate specificity of PDEs was suggested to depend on the ability to rotate of one glutamine which forms hydrophobic bonds with cAMP or cGMP [9, 10]. The fixed glutamine orientation allows to bind only one substrate [11]. It was determined by Zoraghi et al. that Gln-817 is critical for cGMP, sildenafil, and vardenafil affinity. The analogous atoms to substituents at N-1 and C-6 in cGMP form a bidentate H-bond with Gln^817^ [11]. PDE5 was first identified in rat lung tissue, followed by numerous other tissues. The enzyme was first purified and cloned in 1980 [12].

### 1.2. Chemical Structure of Sildenafil

Chemically, sildenafil is a 5-[2-ethoxy-5-(4-methylpiperazin-1-yl)sulfonylphenyl]-1-methyl-3-propyl-1,6-dihydro-7H-pyrazolo[4,3-d]-pyrimidin-7-one. The crystallographic examination of PDE5A shows that the PDE Q pocket accommodates the pyrazolopyrimidinone group of sildenafil. The ethoxyphenyl group of sildenafil fits into the hydrophobic H pocket. The L region of PDE5A surrounds the methylpiperazine group of sildenafil [3, 7].

Orally dosed sildenafil has an expected onset of action of 30 min, with estimated maximum effect at 1 h and a total duration of effect of 4–6 h. In blood, approximately 96% of sildenafil is protein-bound with the pick serum concentration (*C*_max_) of 440 ng/mL being reached at median time of 60 min following administration of a 100 mg oral dose [13].

### 1.3. Sildenafil: Mechanism of Action

Sildenafil increases cellular cGMP levels through competition for the phosphodiesterase binding site with cGMP in that way inhibiting degradation of cGMP to GMP. cGMP through protein kinase G (PKG) plays an important role in the regulation of the activity of various cell populations including immune cells. PKG is abundant in all smooth muscle cells and platelets, and its low levels occur in renal cells, fibroblasts, leukocytes, and neuronal cells. Biological substrates for PKG include inositol, triphosphate receptor (IP_3_R), G-proteins, dopamine- and cAMP-regulated phosphoprotein (DARP-32), and phospholipase C. PKG promotes the opening of calcium-activated potassium channels and activates Ca^2+^ influx, leading to cell hyperpolarization and relaxation [14]. The level of cGMP is also regulated by nitric oxide (NO). NO exerts multiple modulating effects on inflammation and plays a key role in the regulation of the immune responses. Both the inducible nitric oxide synthase (iNOS) and the PDEs enzymes are expressed in numerous cell types including macrophages, dendritic cells, T cells, and neutrophils [15, 16]. NO is generated by three nitric oxide synthase (NOS) isoforms: neuronal (nNOS), endothelial (eNOS), and inducible (iNOS). The nitric oxide is the main activator of soluble guanylate cyclase (sGC), an enzyme which synthesizes guanosine 3′5′-cyclic monophosphate (cGMP). Inhibition of cGMP catabolism by selective PDE5-Is increases NO levels by increasing the ratio of nitrite to nitrate and by stimulating transcription of mRNA for iNOS, as well as eNOS [17].

In addition, nitric oxide increases in tissues and cells the level of heme oxygenase HO-1 which oxidatively degrades heme into equimolar amounts of CO, biliverdin, and iron. Carbon monoxide activates sGC and elevates cGMP in target tissues, which dilates blood vessels. Through that stimulation, sildenafil may serve a critical growth of cellular cGMP levels [18].

Liu et al. [17] demonstrated that sildenafil stimulates the expression of HO-1 and iNOS in vascular smooth muscle cells (SMCs) via the reactive oxygen species-nuclear erythroid 2-related factor 2 (ROS-Nrf2) and sGC-cGMP pathways, respectively [17]. Transcription factor Nrf2 is responsible for the induction of phase II enzymes of xenobiotics metabolism. It activates transcription of genes encoding cytoprotective proteins which deactivates electrophiles metabolites and reactive oxygen species and stabilizes the redox potential of the cell. Nrf2 controls antioxidant defense genes including HO-1, NAD(P)H:quinone oxidoreductase 1 (NQO1), gluthatione reductase (GR), and gluthatione peroxidase (GPx) [18, 19].

Recent data published by de Santana Nunes et al. [20] indicate direct effect of sildenafil on cells. Sildenafil treatment of astrocytes prevents and also restores effect of LPS on actin filaments. Changes in actin stress fibres is a result of overactivation of Ca^2+^ signaling by LPS. de Santana Nunes et al. proposed that sildenafil directly influences cell integrity through ankyrin B, a protein which is associated with the cytoskeleton, and interacts with Na^+^/K^+^-ATP-ase and IP_3_R connecting the pump to the Ca^2+^ responses from internal cell stores and to the integrity of the cytoskeleton. These data show that cGMP is involved in Na^+^/K^+^-ATP-ase activity. Sildenafil acting through Na^+^/K^+^-ATP-ase could change Ca^2+^ waves and stop the inflammatory process.

The action of sildenafil is amplified through these mechanisms.

## 2. Studies Performed on Animals

### 2.1. The Effect of Sildenafil on the Immune System of Healthy Experimental Animals

#### 2.1.1. Adaptive Immune Response

There is some evidence that immunomodulatory properties of sildenafil are gender-specific. In healthy mice, a tendency to decrease the percentage of CD4^+^ cells and to increase the percentage of CD8^+^ T cells was demonstrated in males but not females treated with sildenafil [21]. Karakhanova et al. [21] observed that the levels of T effector memory cells and T central memory cells were decreased in males and increased in females. Naive T cell levels were increased in males, while CD8^+^  T_cm_ (T central memory) cells were decreased. Interestingly, there was no effect on lymphocyte regulatory T cells (Treg) or natural killer T-cells (NKT) in the entire gender-mixed population. The percentage of the activated NK cells and T conventional cells was increased in the female population and decreased in males. Sildenafil treatment did not affect the percentage of dendritic cells in the whole mouse population. SC in healthy mice led to a decrease in percentage of these cells in the spleen of female mice. Moreover, sildenafil was found to diminish serum levels of IL-6 in mice and demonstrated a tendency to increase IL-2. No influence of sildenafil on serum levels of IL-10, IL-1*β* or vascular endothelial grow factor (VEGF) was observed. Sildenafil revealed a considerable immunosuppressive effect in male as opposed to female mice [21]. The differences in immunological effects of the drug between females and males could be explained by endocrine and genetic differences between the sexes [22]. It should be noted that sildenafil in this study was used in large doses—20 mg/kg for 21 days. Ex vivo studies of the effects of sildenafil on isolated splenocytes from healthy mice confirmed a lack of influence on the cytotoxicity of mononuclear cells (CD8^+^, NKT, and NK cells). SC did not affect either maturation or activation of dendritic cells (DC). But with respect to naive/memory phenotype of T cells sildenafil reduced the amount of CD8^+^ cells in whole population. Sildenafil at the concentration of 7.5 *μ*M after 24 h of culture increased the percentage of CD4^+^ T cells and reduced the percentage of B cells [21].

Szczypka and Obmińska-Mrukowicz [23] investigated the effects of sildenafil on thymocytes, splenocytes, and T cells isolated from mice lymph nodes. Oral administration of sildenafil at a dose 1 mg/kg temporarily decreased the percentage of CD4^+^CD8^+^ thymocytes and increased CD8^+^ cells. This effect was only observed after the fifth dose of the drug administrated at 24 h intervals. Among lymphocytes isolated from the mesenteric lymph nodes, the percentage of CD19^+^ cells decreased and a rise in the percentage of CD3^+^ cells was noted 72 h after the last dose of the drug. In another study researchers used the same protocol of administration of SC to investigate cytokine concentration in animals serum. Sildenafil decreased IL-2 level after 72 h from a single administration of 1 mg/kg and after 12 h enhanced temporarily the level of IL-5 (from 7 to 13 pg/mL) [24].

#### 2.1.2. Innate Immune Response

Szczypka and Obmiska-Mrukowicz [25] showed that sildenafil increased the production of IL-1*β* and nitric oxide (NO) by peritoneal macrophages ex vivo, increased the percentage of phagocytosing granulocytes, and decreased the percentage of phagocytosing monocytes.

### 2.2. The Effect of Sildenafil on the Experimental Animals Immune System in Pathological Conditions

#### 2.2.1. Adaptive Immune Response

PDE5 is present in glial cells and neurons [26, 27]. Trapp and Nave [28] and Prado et al. [29] reported that immunomodulatory therapies can be particularly beneficial in the treatment of multiple sclerosis (MS), a disease in which the immune system reacts against the central nervous system (CNS) antigens, initiating a detrimental inflammatory cascade which leads to demyelination and axonal degeneration. Pifarré et al. [30], in their research on experimental autoimmune encephalomyelitis (EAE) as a model of MS, found that administration of sildenafil to mice (10 mg/kg, equivalent to a dose of 57 mg/day for a 70-kg human) for 7 days, at the onset of clinical symptoms, resulted in reduced cellular infiltration in spinal cord and white matter. Sildenafil treatment increased the number of cells expressing the forkhead box p3 (Foxp3) transcription factor, as well as the expression of mRNA of granzyme B (GrB) cluster proteins. CD4^+^ and CD8^+^ Treg cells are known to act by granzymes in the inhibition of T effectors cells and antigen presenting cells (APC). It was shown that GrB protein was upregulated in Treg cells [30]. Moreover, production of IL-2, IFN-*γ*, and IL-4 in cultures of splenocytes isolated from sildenafil-treated mice was reduced. A tendency to reduce the level of TNF-*α* and IL-17 was also observed. Sildenafil did not change the level of inhibitory cytokine IL-10. In vitro proliferation of splenocytes from control group mice after stimulation with phytohemagglutinin (PHA) and exposure to sildenafil from 0.1 to 10 *μ*M/mL was decreased in the SC concentration-dependent manner [30]. Examination of sildenafil-treated EAE mice brains showed that infiltration of inflammatory cells to the brain was reduced due to a lower expression of the intracellular adhesion molecule-1 (ICAM-1). It was found that sildenafil also decreased the level of autoantibodies against myelin oligodendrocyte glycoprotein in the serum [31].

A study by Nunes et al. [32] confirmed a strong anti-inflammatory effect of sildenafil as a result of influence on TNF-*α*, IFN-*γ*, IL-2, and IL-1*β* production in cerebella in sildenafil-treated MS mice. Nunes et al. continued their research [33] in cuprizone- (CPZ-) induced demyelination in mice. Mice treated with sildenafil and CPZ for 15 days had decreased levels of IL-1*β* and TNF-*α* in the serum compared to the CPZ group, but sildenafil did not affect concentration of IL-2. Expression of NO was significantly increased in animals treated with sildenafil, in comparison to the control group. Expression of IL-10 was higher in cerebella from sildenafil-treated mice than in CPZ group.

Research by Luna et al. [34] on placental and trophoblast cells from LPS-induced abortion mice provided insights into protective properties of sildenafil during pregnancy. Orally administered sildenafil blocks transcription of nuclear factor-*κ*B (NF-*κ*B) in nuclei of trophoblast cells and decreased the level of proinflammatory cytokines TNF-*α* and IL-1*β* in placenta fragments. P-Selectin (P-Sel) expression was analyzed in the spongiotrophoblast area, where giant trophoblastic cells are predominant. The LPS-treated group had decreased expression of P-Sel. Sildenafil either alone or in combination with heparin was beneficial for maintaining higher levels of P-Sel, similar to what was found in the control group [35]. Adhesion molecules such as P-selectin play a role in embryo implantation and placentation and can also be a marker of healthy placentas [36, 37].

Serafini et al. [38] and Meyera et al. [39] found that in tumor-bearing mice the inhibition of PDE5 with sildenafil prolongs survival of the animals through the augmentation of antitumor immunity. This effect was achieved due to the inhibition of myeloid-derived suppressor cells (Gr-1^+^CD11b) and downregulation of IL-4R*α*. Consequently, it resulted in the restoration of the CD8^+^ T cell response.

#### 2.2.2. Innate Immune Response

Sildenafil is known to improve the effect of NO. NO-cGMP pathway has neuroprotective and antiapoptotic effects by increasing the level cGMP. Intracellular accumulation of cGMP in different models of inflammation reduces production of proinflammatory cytokines and reduces oxidative stress [25].

The study by Yildirim et al. [40] aimed to investigate the possible protective effects of sildenafil citrate on tissue integrity, oxidant–antioxidant status, and neutrophil infiltration to the inflamed organ in a rat model of bleomycin-induced lung fibrosis. Treatment of animals with sildenafil (10 mg/kg subcutaneously for 14 days) was beneficial with regard to prevention of lipid peroxidation, cytokine (IL-1*β* and TNF-*α*) production, neutrophil accumulation, and myeloperoxidase (MPO) activation.

Zhao et al. [41] showed that sildenafil exerts anti-inflammatory effects in vitro in LPS-activated microglial cells by blocking the nuclear factor kappa-light-chain-enhancer of activated B cells (NF-*κ*B) and Mitogen Activated Protein Kinase (MAPK) activation. Sildenafil markedly inhibited iROS production induced by LPS, which may be partly due to MAPKs potent downregulation of the NADPH-derived iROS production [40].

Pifarré et al. [42] found that sildenafil treatment in a murine model of EAE resulted in changes in the expression of several genes implicated in responses to stress and inflammation, as well as genes involved in cytoskeleton reorganization and wound healing. Sildenafil caused 2.5-fold increase of mRNA expression of inflammation-related genes chitinase 3-like 3 (YM-1) protein secreted by peritoneal macrophages. On the other hand, microglia/macrophages ionized calcium-binding adapter molecule-microglia activation marker (Iba-1) was decreased. These results suggest that sildenafil promotes a switch from the M1 proinflammatory (M1) to the anti-inflammatory (M2) phenotype macrophages [42]. Macrophages generated from monocytes can differentiate into classically activated M1 or alternatively activated M2. M2 macrophages promote wound healing and secrete TGF-*β*, an immunoregulatory and anti-inflammatory cytokine [43, 44]. These results indicate that sildenafil induces a shift from the classical to the alternative microglia/macrophage phenotype which has been associated with neuroprotective functions that promote repair processes and wound healing [44].

Further research on neurodegenerative diseases models by Raposo et al. [45] demonstrated that sildenafil reduces the expression of cytokines as well as cyclooxygenase-2 (COX-2) and astrocyte activation marker (GFAP) in a demyelinating model induced in wild-type (WT) mice. Sildenafil reduced expression of Iba-1 and increased concentration of IK*βα* - NF*κ*B inhibitory protein in activated M1 macrophages. The administration of sildenafil decreased expression of NF*κ*B, GFAP, inactivated AMP-activated protein kinase (AMPK), and iNOS. AMPK is an intracellular energy sensor that plays central role in glucose and lipid metabolism. It also downregulates inflammation in vitro and in various animal models. NO may act as an endogenous activator of AMPK and in an opposite way inactivates NF*κ*B by phosphorylation of inhibitor k binding *α* (IK*βα*). The study proved that sildenafil exerts its anti-inflammatory effect probably through AMPK-eNOS/NO-NF*κ*B signaling. Sildenafil can also act directly through cGMP and indirectly by PKA on NF*κ*B inhibition [45]. Administration of sildenafil reduced the expression of IL-1*β* and TNF-*α* and increased the level of anti-inflammatory cytokine IL-10 [41, 45].

There is ample evidence to indicate the role of CCR-2 (monocyte chemoattractant protein 1-MCP-1-receptor-CCl-2) in tissue repair after injury and increased neurogenesis due to its chemotactic property in neural precursor cells [46]. Nunes et al. [47] suggested that increase of CCR-2/MCP-1 is associated with the stimulation cGMP-PKG signaling induced by sildenafil, and it can stimulate protective M2 phagocytic phenotype of microglia. This study also demonstrated that expression of extracellular metalloproteinase-9 (MMP-9) is increased after sildenafil treatment. Most MMPs are secreted as inactive proteins which are activated when cleaved by extracellular proteinases. However, it has been proposed that MMPs have a role in modulating different physiological processes, such as reproduction, angiogenesis, bone development, apoptosis, and cell migration. Nunes et al. reported that sildenafil facilitated vascular remodeling by enhancement of MMP-9 expression [47].

Lung ischemia/reperfusion (I/R) injury plays an important role in outcome of organ transplantation. Results obtained by Pizanis et al. [48] in studies on rats suggested that watering with saline and sildenafil (10 mg/kg) 3 h before the operation improved lung I/R injury by decreasing the production of IL-6 and TNF-*α*. It has been shown that pretreatment with sildenafil normalizes TNF-*α* and IL-6 levels in lung tissue, which suggests that pretreatment with sildenafil alleviated inflammation in early reperfusion injury.

Similar findings were reported recently by Zahran et al. [49], after investigation of rats renal ischemia/reperfusion injury. Watering rats orally with SC 1 mg/kg 60 min before anesthesia resulted in activation of antioxidant genes (Nrf2, HO-1 and NQO-1-quinone oxidoreductase) and antiapoptotic gene (Bcl-2) and attenuation of proinflammatory cytokines (TNF-*α*, IL-1*β*) as well as ICAM-1. Expression of genes was evaluated in ischemic kidney tissue after nephrectomy. Effects of sildenafil were maximal after 2 days from SC administration.

## 3. Studies Performed in Humans

### 3.1. The Effects of Sildenafil on the Lymphocytes Subpopulations of Healthy Human Blood Donors

The only data of the effects of sildenafil on healthy human lymphocytes in vitro comes from Pifarré et al. [30] studies on human T regulatory cells.Cocultures of T effector cells and T reg cells (0.25 : 1 and 0.5 : 1 ratio) isolated from healthy donor peripheral blood showed that sildenafil at the 10 *μ*M concentration influenced the ability of Tregs to downregulate T effector cell proliferation. Moreover, expression of Treg transcription factor Foxp3 was increased, suggesting that upregulation of Tregs is involved in T effector cell deactivation.

### 3.2. The Effects of Sildenafil on the Immune System in Patients Treated with Sildenafil

The influence of sildenafil on the TNF-*α* level, Treg, and NK activity in patients with recurrent abortion was reported. Jerzak et al. [50] found that sildenafil significantly reduced peripheral blood NK activity in 38 women with natural and after in vitro fertilization (IF) recurrent abortion. Sildenafil was administered intravaginally during the proliferative phase of the menstrual cycle for 3 or 6 days. NK cells activity was also investigated in in vitro cultures of mononuclear cells (MNC) of the patients and the control group. Sildenafil added at the 10 *μ*g/mL concentration to the culture of MNC of healthy women reduced NK cell activity [50]. Determination of serum TNF-*α* level revealed a tendency to increase after sildenafil therapy [51]. These results were in contrast with the findings of El-Far et al. [52]. In the latter study, the percentages of CD3^+^CD56^+^CD161^+^ NKT cells and TNF-*α* positive T cells were greatly reduced after sildenafil intravaginal administration (25 mg/4 times a day for 24 days). The differences could be explained by different dosing of the drug.

Preeclampsia is a disorder of pregnancy characterized by high blood pressure and level of protein in urine, due to endothelial dysfunction and is a primary cause of maternal morbidity. It affects 2–8% of pregnancies worldwide, and, if not treated, leads to eclampsia. Complications include aspiration pneumonia, cerebral hemorrhage, kidney failure, and cardiac arrest [53]. The vascular endothelial grow factors (VEGFs) family of proteins and their receptors are thought to be key contributors to this disease. It is believed that high level of VEGF and placental growth factor (PLGF) are involved in vascular remodeling of cytotrophoblast. The VEGF-receptor-1 is denoted as soluble fms-like tyrosine kinase-1 (sFlt-1) as it belongs to fms related tyrosine kinase family. Placenta of pregnant women with preeclampsia produces high levels of sFlt-1. sFlt-1 binds to free VEGF and PLGF, inactivates them, and makes them unavailable for proper signaling [54]. Advanced glycation end-products (AGEs) represent the molecular complexes generated as a result of nonenzymatic reactions of carbohydrates and oxidized lipid with proteins. These reactions lead to the irreversible cross-linking of proteins and, as a consequence, loss of protein structure and function. Previous studies demonstrated that serum AGEs in preeclamptic women were significantly higher than in healthy women. Accumulation of AGEs may induce oxidative injury and vascular perturbation in placental bed, leading to preeclampsia [55]. The study of Jeong et al. showed that AGEs contribute to elevation of sFlt-1. The study was conducted on JEG-3 cell line established from choriocarcinoma that was shown to retain trophoblastic cell-like characteristics. They observed that AGEs-BSA increased sFlt-1 mRNA expression and protein release as well as increasing the production of ROS and NF-*κ*B in JEG-3. Sildenafil citrate suppressed sFlt-1 mRNA expression and protein release in cells treated with AGEs-Bovine Serum Albumin in a dose-dependent manner (concentration range 5–100 *μ*mol/mL) [56].

Unexpected results of healing of one patient from B-cell chronic lymphocytic leukemia (B-CLL) treated only with sildenafil were reported by Sarfati et al. [57]. During a 3.5-year therapy with sildenafil (50 mg once a week), the patient lymphocyte count decreased from 20 × 10^9^ to 3 × 10^9^. In addition, sildenafil induced apoptosis of the B-CLL cells in caspase 3-dependent way in vitro. It has been shown that IL-4 abrogated the effect of sildenafil. Interestingly, there was no killing effect of sildenafil on normal B-cells.

Treon et al. [58] reported similar findings in 5 patients suffering from Waldenstrom's macroglobulinemia (WM) and erectile dysfunction. One patient had complete remission of WM. WM tumor cells were also culture-sorted with sildenafil at pharmacologically relevant levels and apoptosis was demonstrated in tumor cells from all 5 patients. Possibly, the modulating effect of sildenafil on lymphocyte subpopulations and humoral immune responses is also mediated by the synthesis and release of cytokines [21, 22, 59].

Sildenafil improves the condition of patients with Reynaud's phenomenon and helps wound healing in scleroderma by inhibiting the TGF-*β*–Rho kinase pathway. TGF-*β* acts as an antiproliferative factor in normal epithelial cells and at the early stages of oncogenesis [60, 61]. Some cells which secrete TGF-*β* also have receptors for this cytokine, a phenomenon known as autocrine signaling. Cancer cells increase production of TGF-*β*, which also affects the surrounding cells. The Rho GTPases are involved in numerous signal transduction pathways and act as regulators of the actin cytoskeleton, cell motility, and transcription. They are associated with progression to malignancy in several types of cancer. The Rho GTPases are molecular switches which remain inactive when GDP bound and active when GTP bound, and they expand their signals through interaction with numerous downstream signaling effectors [62–64]. In this way it is possible that sildenafil affects oncogenesis.

Guilluy et al. [65] suggested that sildenafil inhibits RhoA/Rho kinase-dependent functions in the pulmonary artery through enhanced RhoA phosphorylation and cytosolic sequestration by GDP. The inhibition of intracellular events downstream of RhoA thus participates in the beneficial effect of sildenafil in pulmonary hypertension.

Caravita et al. [66] described a patient with multiple sclerosis who developed severe PAH after treatment with IFN-*β*-1a. Sildenafil reversed the detrimental effect of IFN-*β* on the cardiovascular system.

Reduced NO production is correlated with poor wound healing in diabetic patients. Sildenafil improved the production of NO in diabetic subjects. Chronic administration of sildenafil in diabetic patients was associated with an increase in nitrite/nitrate levels and improved markers of vascular inflammation with a decrease of endothelin-1, IL-6 level, lower expression of integrins, including intracellular adhesion molecules (ICAM), and vascular adhesion molecules (VCAM). Furthermore, the effect of sildenafil therapy was sustained after one month from withdrawal of the drug [67, 68].

### 3.3. Concluding Remarks

A growing body of evidence shows that sildenafil exerts immunomodulatory effects. Its positive action was demonstrated in treating severe autoimmune diseases and cancer. In addition, anti-inflammatory and antiaggregation effects of sildenafil have also been reported. In tumor-bearing mice, the inhibition of PDE5 activity with sildenafil prolonged the survival of the animals through augmentation of antitumor immunity. Daily sildenafil treatment from EAE symptom onset prevented further clinical deterioration and improved neurogenesis. Sildenafil decreased the levels of proinflammatory cytokines, including TNF-*α*, IL-1, and reduced NK cells activity, and enhanced the action of regulatory T cells. Sildenafil markedly inhibited iROS production induced by LPS. Type 5 phosphodiesterase (PDE5) inhibitor increased endothelial cell cGMP and promoted angiogenesis by increasing the expression of VEGF. De novo blood vessel formation is essential for embryonic vascular development and for postnatal vascular homeostasis and wound healing.

Thus, the available data suggest that sildenafil could find use in the treatment of autoimmune, neurodegenerative, and cardiovascular diseases, as well as recurrent abortions. However, the potential immunomodulatory effects of sildenafil in humans remain to be confirmed.

## Abbreviations

B-CLL: B-cell chronic lymphocytic leukemia
cGMP: Cyclic guanosine monophosphate
ED: Erectile dysfunction
GMP: Guanosine monophosphate
IL: Interleukin
NKT: Natural killer T-cells
NO: Nitric oxide
NOS: Nitric oxide synthase
PDE-Is: Phosphodiesterase inhibitors
PDEs: Phophodiesterases
PKG: Protein kinase G
ROS-Nrf2: Reactive oxygen species-nuclear erythroid 2-related factor 2
SC: Sildenafil citrate
sGC: Soluble guanylate cyclase
TGF-*β*: Transforming growth factor-*β*
TNF-*α*: Tumor necrosis factor-*α*
Treg: Lymphocyte T regulatory cells.
